# Delineated 3-1-BenCarMethInYlPro-Phosphonic Acid’s Adroit Activity against Lung Cancer through Multitargeted Docking, MM\GBSA, QM-DFT and Multiscale Simulations

**DOI:** 10.3390/ijms25010592

**Published:** 2024-01-02

**Authors:** Mohammed Ageeli Hakami, Ali Hazazi, Fawaz Albloui, Amal F. Gharib, Fouzeyyah Ali Alsaeedi, Osama Abdulaziz, Abdulfattah Y. Alhazmi, Ahad Amer Alsaiari

**Affiliations:** 1 Department of Clinical Laboratory Sciences, College of Applied Medical Sciences, Shaqra University, Al-Quwayiyah 19257, Saudi Arabia; m.hakami@su.edu.sa; 2 Department of Pathology and Laboratory Medicine, Security Forces Hospital Program, Riyadh 11481, Saudi Arabia; ahazazi@sfh.med.sa (A.H.); falbloui@sfh.med.sa (F.A.); 3 Department of Clinical Laboratory Sciences, College of Applied Medical Sciences, Taif University, P.O. Box 11099, Taif 21944, Saudi Arabia; amgharib@tu.edu.sa (A.F.G.); fouzeyyah@tu.edu.sa (F.A.A.); o.osama@tu.edu.sa (O.A.); 4 Pharmaceutical Practices Department, College of Pharmacy, Umm Al-Qura University, Makkah 21955, Saudi Arabia; ayhazmi@uqu.edu.sa

**Keywords:** lung cancer, multitargeted drug designing, pharmacokinetics, molecular interaction fingerprints, molecular dynamics simulation

## Abstract

Lung cancer is a pervasive and challenging disease with limited treatment options, with global health challenges often present with complex molecular profiles necessitating the exploration of innovative therapeutic strategies. Single-target drugs have shown limited success due to the heterogeneity of this disease. Multitargeted drug designing is imperative to combat this complexity by simultaneously targeting multiple target proteins and pathways, which can enhance treatment efficacy and overcome resistance by addressing the dynamic nature of the disease and stopping tumour growth and spread. In this study, we performed the molecular docking studies of Drug Bank compounds with a multitargeted approach against crucial proteins of lung cancer such as heat shock protein 5 (BIP/GRP78) ATPase, myosin 9B RhoGAP, EYA2 phosphatase inhibitor, RSK4 N-terminal kinase, and collapsin response mediator protein-1 (CRMP-1) using HTVS, SP with XP algorithms, and poses were filtered using MM\GBSA which identified [3-(1-Benzyl-3-Carbamoylmethyl-2-Methyl-1h-Indol-5-Yloxy)-Propyl-]-Phosphonic Acid (3-1-BenCarMethIn YlPro-Phosphonic Acid) (DB02504) as multitargeted drug candidate with docking and MM\GBSA score ranges from −5.83 to −10.66 and −7.56 to −50.14 Kcal/mol, respectively. Further, the pharmacokinetic and QM-based DFT studies have shown complete acceptance results, and interaction fingerprinting reveals that ILE, GLY, VAL, TYR, LEU, and GLN were among the most interacting residues. The 100 ns MD simulation in the SPC water model with NPT ensemble showed stable performance with deviation and fluctuations <2 Å with huge interactions, making it a promising multitargeted drug candidate; however, experimental studies are needed before use.

## 1. Introduction

Lung cancer remains a formidable global health challenge, characterised by its pervasive nature and complex molecular profiles. The limited success of single-target drugs in treating this disease underscores the urgent need for innovative therapeutic strategies [1,2]. This necessity arises from the heterogeneity of lung cancer, a multifaceted condition presenting diverse molecular alterations that make it resistant to one-size-fits-all treatments. A paradigm shift towards multitargeted drug design is essential to combat this complexity. Such an approach simultaneously targets multiple proteins and pathways in lung cancer, potentially enhancing treatment efficacy and overcoming resistance mechanisms [3]. The following introduction delves into the intricate landscape of lung cancer, emphasising the critical role of multitargeted drug design in tackling this formidable disease. Lung cancer has earned its reputation as one of the most pervasive and challenging diseases worldwide. It is the leading cause of cancer-related mortality, responsible for more deaths than breast, prostate, and colon cancers combined [4,5,6,7]. The disease’s pervasiveness knows no boundaries, affecting men and women across diverse populations and age groups. Despite extensive research efforts and advancements in medical science, lung cancer poses a significant public health burden. One of the defining characteristics of lung cancer is its complex molecular landscape [8,9,10]. Unlike some other cancers, lung cancer is not a monolithic disease. Instead, it encompasses a spectrum of subtypes associated with distinct genetic alterations and molecular signatures. These variations contribute to the disease’s heterogeneity and pose a significant challenge to effective treatment. Historically, cancer treatment has relied heavily on single-target drugs to inhibit specific proteins or pathways in tumour growth. While these drugs have shown promise in some cancer types, their efficacy in lung cancer has been limited. The reasons for this limited success are multifaceted. Firstly, the molecular diversity of lung cancer means that a single-target drug may only be effective for a subset of patients whose tumours harbour the specific target [11,12,13,14,15]. In essence, these drugs often fail to account for the heterogeneous nature of the disease. Secondly, lung cancer is notorious for its ability to develop resistance to single-target therapies. Tumour cells can adapt and evolve, rendering these drugs ineffective over time. This adaptability is a significant obstacle in lung cancer treatment and highlights the need for more comprehensive therapeutic approaches [16,17,18,19,20].

A paradigm shift towards multitargeted drug design is imperative to the complexity and heterogeneity of lung cancer. This innovative approach seeks to develop drugs simultaneously targeting multiple proteins and pathways critical to tumour growth and survival. By doing so, multitargeted drugs have the potential to enhance treatment efficacy and overcome resistance mechanisms. Within the multifaceted realm of lung cancer, proteins are critical players in maintaining cellular integrity and suppressing tumour growth [21,22,23,24]. These proteins are critical regulators of various cellular processes and are intricately involved in tumour suppression mechanisms. The first protein is human 70 kDa Heat Shock Protein 5 (BIP/GRP78) ATPase Domain, also known as BIP or GRP78, is a molecular chaperone that assists in protein folding and assembly, which often upregulated in cancer cells and is associated with tumour survival and resistance to therapy [25]. The second protein is the human myosin 9B RhoGAP domain, which is involved in cellular motility and cytoskeleton regulation, and its role in lung cancer may relate to its effects on cell migration and invasion, which are crucial aspects of tumour progression [26]. The third protein is the EYA2 phosphatase inhibitor, a phosphatase that plays a role in DNA repair and transcriptional regulation, and its inhibitors may have potential as anti-cancer agents, particularly in cases where EYA2 is overexpressed [27]. The fourth protein, RSK4 N-terminal kinase domain, is a kinase that can regulate various cellular processes, including cell proliferation and differentiation, and understanding its role in lung cancer could provide insights into tumour growth and potential therapeutic targets [28]. The fifth protein is human collapsin response mediator protein-1 (CRMP-1), which is associated with axonal guidance and neuronal development, and in lung cancer, it has been identified as a potential tumour suppressor [29]. These proteins represent critical nodes in the intricate network of molecular interactions governing lung cancer. Their roles in structural maintenance and tumour suppression make them promising candidates for targeted therapy.

This study aims to identify a multitargeted drug candidate that potentially can target all five proteins and have an excellent binding score and stable performance. We conducted the docking studies with HTVS, SP, and XP algorithms and filtered the docked poses with MM\GBSA [1,30,31,32]. Further, we have also performed the pharmacokinetics computations and molecular interactions fingerprints to understand the pattern and the MD simulation to evaluate all the results and check if the compounds are stable and can be validated experimentally in the future or not.

## 2. Results

### 2.1. Protein Structural Analysis

**Figure 1:**
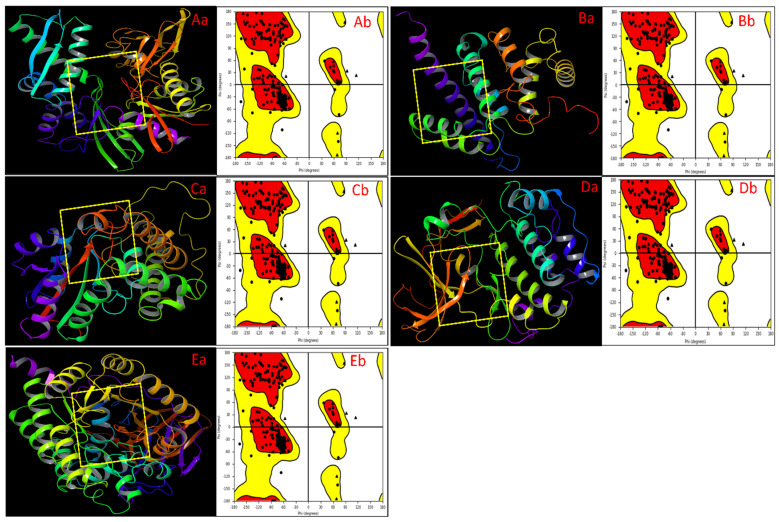
Showing the prepared 3D structures of the proteins and ligand binding sites of (**Aa**) 3IUC, (**Ba**) 5C5S, (**Ca**) 5ZMA, (**Da**) 6G77, and (**Ea**) 4B3Z. The Ramachandran plot for the final prepared proteins shows the structures are entirely acceptable for each case of (**Ab**) 3IUC, (**Bb**) 5C5S, (**Cb**) 5ZMA, (**Db**) 6G77, and (**Eb**) 4B3Z.

In our study, we meticulously prepared and validated protein structures for molecular docking, employing a comprehensive methodology that included analysing Ramachandran plots. We commenced by retrieving 3D structures of target proteins with PDBID: 3IUC, 5C5S, 5ZMA, 6G77, and 4B3Z from the RCSB Protein Data Bank [25,26,27,28,29]. These structures underwent meticulous pre-processing within Maestro’s Protein Preparation Workflow (PPW), including side chain completion, loop modelling, bond order assignment, hydrogen removal, and disulfide bond formation, ensuring structural integrity, and the final figures are shown in Figure 1. The validation process was exemplified by the analysis of Ramachandran plots, which assessed dihedral angles of amino acid residues, confirming their placement within the favourable regions of the plot. This analysis provided critical assurance of the structural correctness and suitability of these proteins for subsequent molecular docking studies, bolstering the foundation of our study. In the Ramachandran plot, the phi (ϕ) and psi (ψ) angles represent the dihedral angles of amino acid residues in a protein’s backbone. These angles are crucial for understanding a protein’s conformational space and structural stability. The phi angle refers to the rotation around the N-Cα bond, which is the bond between an amino group (N) and an amino acid residue’s alpha carbon (Cα), and it determines the orientation of the amino acid’s amino group relative to its alpha carbon. The psi angle refers to the rotation around the Cα-C bond, the bond between the alpha carbon (Cα) and an amino acid residue’s carbonyl carbon (C), and it determines the orientation of the amino acid’s carbonyl group relative to its alpha carbon. A good distribution of residues in a Ramachandran plot typically refers to amino acid residues’ ϕ and ψ dihedral angles falling within the allowed regions, and these allowed regions are defined based on steric considerations and the energetically favourable conformations of the backbone (Figure 1). The residue distribution in each case is entirely acceptable, and we checked with the PPW and found no problem. Some protein residues were not in allowed regions due to their natural exhibit flexibility, especially in regions like loops or termini. While these regions might have deviations in the Ramachandran plot, they did not necessarily indicate a problem and were not part of the core regions of the protein that could be a cause for concern.

### 2.2. Protein–Ligand Interaction Analysis

**Figure 2:**
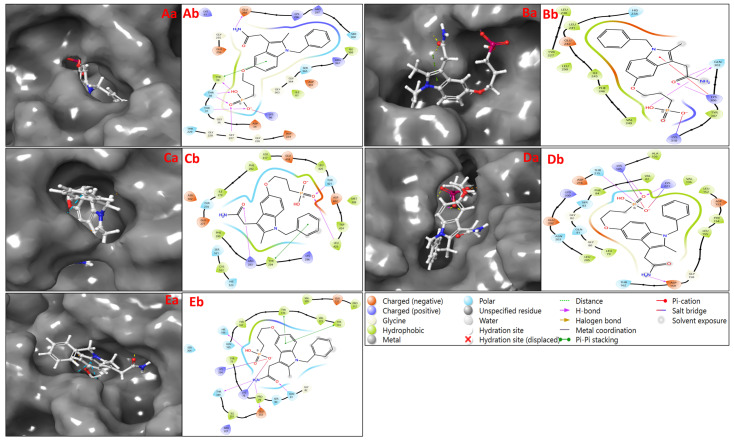
Showing the prepared 3D docked best poses of (**Aa**) 3IUC, (**Ba**) 5C5S, (**Ca**) 5ZMA, (**Da**) 6G77 and (**Ea**) 4B3Z, and 2D ligand interaction diagram of docked poses of (**Ab**) 3IUC, (**Bb**) 5C5S, (**Cb**) 5ZMA, (**Db**) 6G77, and (**Eb**) 4B3Z.

A computational approach to drug development known as “molecular docking” visualises how a tiny molecule binds to the target protein and calculates the strength of the binding affinity. Our study looks at induced fit docking analysis, which names 3-1-BenCarMethInYlPro-Phosphonic acid as the ideal drug candidate. The molecular interaction analysis diagram depicts each adaptive protein structure’s interactions with 3-1-BenCarMethInYlPro-Phosphonic Acid ligands. The interaction of the human 70 kda heat shock protein 5 (BIP/GRP78) ATPase (PDBID: 3IUC) in complex with 3-1-BenCarMethInYlPro-Phosphonic acid has produced a docking score of −9.019 Kcal/mol and an MMGBSA score of −39.68 Kcal/mol (Table 1). While contacting, six hydrogen bonds among GLY227, THR38, and THR37 residues with 2O atoms, TYR39 and THR38 residues along the OH atom, and GLU293 residue along the NH_2_ atom. Additionally, the salt bridge contacts LYS96 residue with O atom and pi–pi stacking along TYR39 residue with the benzene ring of the ligand (Figure 2Aa,Ab). Interaction of the human myosin 9b RHOGAP domain (PDBID: 5C5S) in complex with 3-1-BenCarMethInYlPro-Phosphonic acid has produced a docking score of −5.828 Kcal/mol and MMGBSA score of −36.30 Kcal/mol (Table 1), showing hydrogen bond interactions with GLN303 and LYS306 residues along O atom and GLN303 residue with OH atom. Also, form a salt bridge with LYS310 residue along the O atom and pi–cation along LYS306 residue with the benzene ring of the ligand 3-1-BenCarMethInYlPro-Phosphonic Acid (Figure 2Ba,Bb). The interaction study between allosteric EYA2 phosphatase inhibitor (PDBID: 5ZMA) in complex with 3-1-BenCarMethInYlPro-Phosphonic acid has produced a docking score of −7.471 Kcal/mol and an MMGBSA score of −7.56 Kcal/mol (Table 1) while interacting with hydrogen bonds along ARG293, THR421, and LEU425 residues with 2O atoms and pi–pi stacking contact TYR294 residue with benzene ring of the ligand 3-1-BenCarMethInYlPro-Phosphonic Acid (Figure 2Ca,Cb). The interaction of the RSK4 N-terminal kinase domain (PDBID: 6G77) in complex with 3-1-BenCarMethInYlPro-Phosphonic acid resulted in a docking score of −10.663 Kcal/mol and MMGBSA score of −50.14 Kcal/mol (Table 1), there are four hydrogen bonding between THR215, LYS221, and LYS105 residues along 2O atoms, and ASP159 residue interact with NH_2_ atom. Also, form two salt bridges with LYS105 and LYS221 along the O atom of the ligand (Figure 2Da,Db). The contact between the human collapsin response mediator protein-1 (PDBID: 4B3Z) in complex with 3-1-BenCarMethInYlPro-Phosphonic acid has produced docking score of −9.713 Kcal/mol and MMGBSA score of −43.56 Kcal/mol (Table 1) while interacting hydrogen bonds along GLN81 residue with O atom, HIE198 residue with OH atom, and THR349 and GLU353 residues along NH_2_ atom. Additionally, 2 pi–pi stacking contacts TYR170 and TYR174 residues with the benzene ring; also, they form 2 salt bridges that interact with LYS78 and LYS254 with the O atom of the ligand (Figure 2Ea,Eb). The molecular docking results of 3-1-BenCarMethInYlPro-Phosphonic acid with lung cancer-associated proteins reveal promising interactions. Notably, the complex with the human 70 kDa Heat Shock Protein 5 (BIP/GRP78) ATPase demonstrated a strong docking score supported by hydrogen bonds, salt bridges, and pi–pi stacking interactions. Similarly, the interactions with human Myosin 9b RHOGAP, allosteric EYA2 phosphatase inhibitor, RSK4 N-terminal kinase, and human Collapsin Response Mediator Protein-1 showcased favourable scores and a range of interactions that suggest the potential of 3-1-BenCarMethInYlPro-Phosphonic acid as a multitargeted inhibitor of lung cancer-related proteins, warranting further experimental validation.

### 2.3. Pharmacokinetics and QM-Based DFT Studies

The pharmacokinetic properties of 3-1-BenCarMethInYlPro-Phosphonic acid were computed with the QikProp tool after identifying it as multitargeted docking against cervical cancer proteins and subsequent comparison standard values, providing valuable insights into its drug-like characteristics. Firstly, its small molecular size suggests potential suitability for drug development, and it exhibits 15 ring atoms, indicating a complex yet compact structure. Remarkably, it complies with Lipinski’s Rule of Three, a fundamental guideline for drug-likeness, by having a single violation. This violation arises from its slightly higher molecular weight (416.413 g/mol) than the typical 300 g/mol limit, suggesting good bioavailability. In terms of oral absorption, it receives a high score, with a value of 1 indicating favourable absorption potential. This compound also demonstrates a significant % of human oral absorption (45.275%), which suggests a likelihood of efficient uptake in the body. Importantly, the compound exhibits an acceptable number of hydrogen bond donors (4) and acceptors (8.25), which can facilitate intermolecular interactions crucial for drug activity. Furthermore, several physicochemical properties align well with drug development criteria. It has a relatively low dipole moment (3.074 D), indicating potential for reduced toxicity. The compound’s polarizability (40.839 Å^3^) and solvent-accessible surface area (SASA) (722.945 Å^2^) fall within acceptable ranges. The calculated LogP values for water (QPlogPw) and octanol (QPlogPoct) are within the desired limits, indicating good hydrophilic–lipophilic balance. It exhibits an acceptable globularity index (0.7929173), signifying a compact and well-folded structure. However, it is worth noting that this compound has a negative CNS score (−2), suggesting potential inactivity in the central nervous system. These findings support its potential as a drug candidate against cervical cancer, although further experimental validation is warranted, and the detailed view is presented in Table 2.

**Figure 3:**
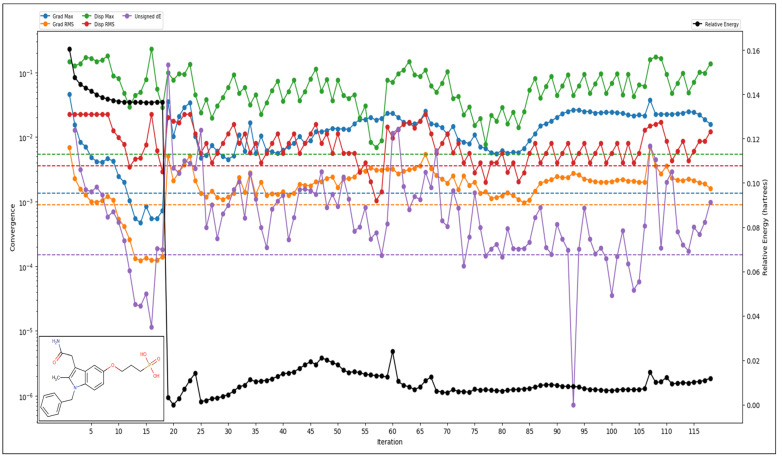
The QM-based Density Functional Theory computations and the results for the various energies are shown, where the relative energy is shown in black.

The analysis of 3-1BCMIYPPA using computational methods has provided detailed insights into its properties. When it comes to its electronic structure, this molecule has 557 canonical orbitals. The HOMO energy level is −0.053769 Hartree, while the LUMO energy level is −0.006937 Hartree. It has a dipole moment of 37.3867 Debye, indicating its overall polarity. In terms of vibrations, the molecule exhibits a range of frequencies, from 25.961 cm^−1^ to 3739.59 cm^−1^, and importantly, no negative frequencies were detected, indicating its stability. The zero-point energy, a measure of its lowest possible energy at absolute zero temperature, is calculated to be 268.71 kcal/mol. Several thermodynamic properties were determined at standard conditions (298.15K and 1.00 atm pressure). The molecule has an entropy of 171.653 Kcal/mol, an enthalpy of 17.239162 kcal/mol, and a free energy of −33.93919 kcal/mol. Its internal energy is 16.646677 kcal/mol, and its heat capacity is 105.347 Kcal/mol/. The natural logarithm of the partition function (ln(Q)) at these conditions is 57.283. Regarding the molecule’s total energy in atomic units, the internal energy is −1641.585286 au, the enthalpy is −1641.584342 au, and the free energy is −1641.6659 au (Figure 3). The electrostatic potential (ESP) analysis provides information about the distribution of electrical charges, with values ranging from −144.99 kcal/mol (minimum) to 2.7 kcal/mol (maximum) and an average of −54.36 kcal/mol. The ESP local polarity is determined to be 27.45 kcal/mol. Additionally, the Atom-Least-Interaction Energies (ALIE) analysis sheds light on noncovalent interactions within the molecule. The minimum ALIE is 67.81 kcal/mol, the maximum is 317.71 kcal/mol, and the average ALIE is 210.95 kcal/mol. On average, the ALIE deviates from the mean by about 41.560254 kcal/mol. These findings provide a comprehensive understanding of 3-1BCMIYPPA’s characteristics and can be valuable for applications in drug design and related research endeavours.

### 2.4. Interaction Fingerprints

**Figure 4:**
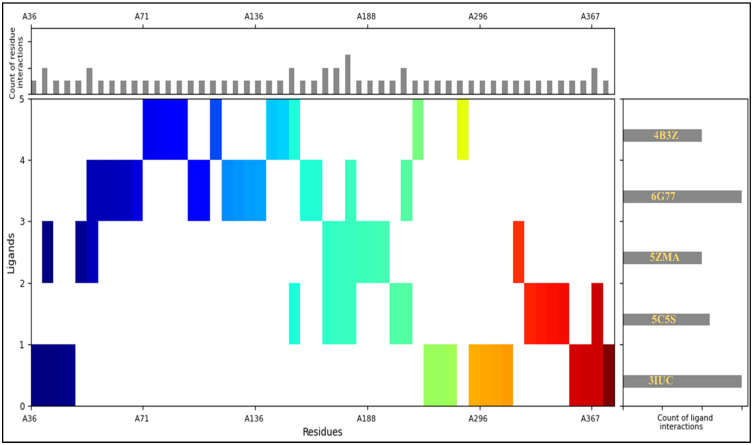
Molecular Interaction Fingerprints where the main-coloured plot shows the N to C terminal of the protein. The right side plot shows the count of ligand interactions, and the upper plot shows the count of residue interactions.

The molecular interaction fingerprints were performed on the docked poses and plotted by residue distribution, ligand interaction count, and residue interactions. The most interactions were found in 6G77 and 3IUC, followed by 5C5S, 5ZMA, and 4B3Z, shown in the right-side plot of the count of ligand interactions in Figure 4. The residue distribution from N to C terminal is blue to red. The most number of contacted residue counts were found of 7ILE, 6GLY, 5VAL, 5TYR, 5LEU, 5GLN, 4GLU, 3THR, 3SER, 3PHE, 3ARG, 2PRO, 2LYS, 2HIE, 2ASP, 2ASN, 2ALA, 1TRP, 1MET, 1HIS, and 1ASP, making the complexes stable on the binding pocket (Figure 4). The stability of the complexes formed between 3-1-BenCarMethInYlPro-Phosphonic acid and the target proteins is underscored by specific residues in the molecular interaction fingerprints. The fingerprints revealed substantial interactions with critical amino acid residues, contributing to the binding pocket’s overall stability. Among these, ILE (7 interactions), GLY (6 interactions), VAL (5 interactions), TYR (5 interactions), LEU (5 interactions), GLN (5 interactions), and GLU (4 interactions) emerge as prominent contributors, demonstrating their pivotal role in anchoring the ligand within the binding site. Additionally, THR, SER, PHE, ARG, PRO, LYS, HIE, ASP, ASN, ALA, TRP, MET, HIS, and ASP residues, each with varying counts, further bolstering the stability of these complexes. These molecular interactions collectively foster a robust and reliable binding interface, affirming the compound’s potential as a potent inhibitor.

### 2.5. Molecular Dynamics Simulations

The system builder has produced 44360 atoms in 3IUC, 37483 atoms in 5C5S, 35396 in atoms 5ZMA, 37935 atoms in 6G77, and 48604 atoms in 4B3Z in the neutralised condition, and after the production run for 100ns MD simulation in SPC water model was performed that has produced 1000 frames by recording at each 100ps and the simulation interaction diagram tool was used for the analysis of RMSD, RMSF, and simulation interaction diagram.

#### 2.5.1. Root Mean Square Deviation (RMSD)

**Figure 5:**
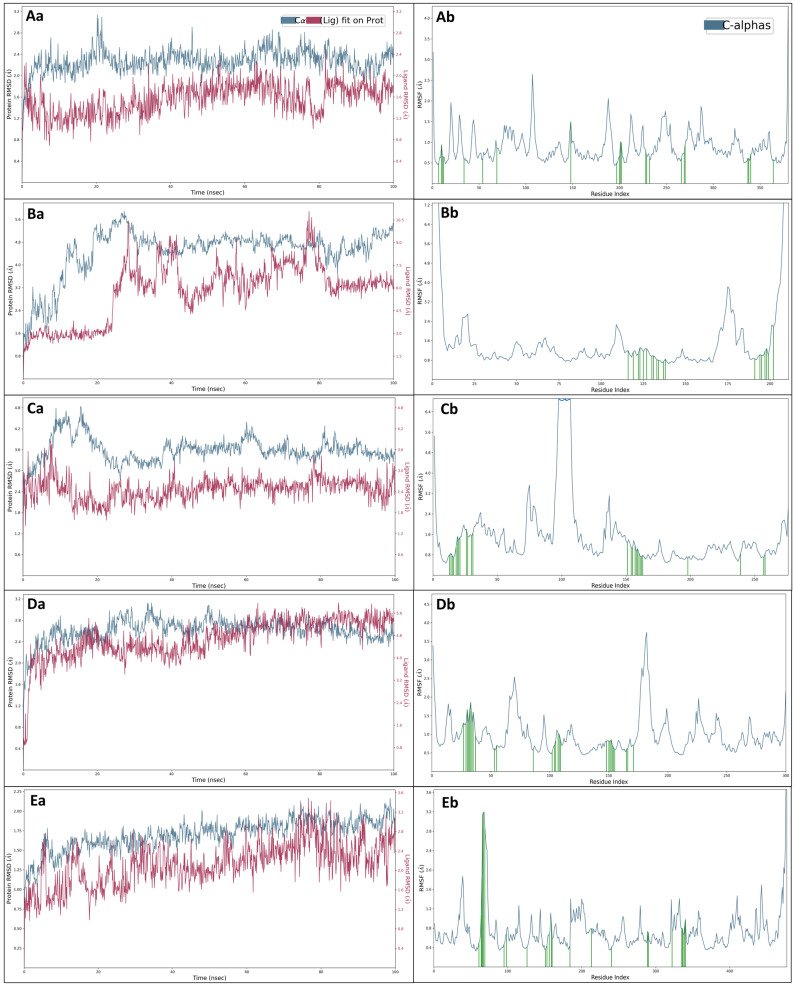
Showing the Root Mean Square Deviation (RMSD) of 3-1-BenCarMethInYlPro-Phosphonic Acid in complex with (blue) (**Aa**) 3IUC, (**Ba**) 5C5S, (**Ca**) 5ZMA, (**Da**) 6G77 and (**Ea**) 4B3Z, and Root Mean Square Fluctuations (RMSF) (blue) of (**Ab**) 3IUC, (**Bb**) 5C5S, (**Cb**) 5ZMA, (**Db**) 6G77, and (**Eb**) 4B3Z and the green lines are showing the 3-1-BenCarMethInYlPro-Phosphonic Acid contact with amino acids of the proteins at shown residue index.

The RMSD is a mathematical measure used in structural biology and computational chemistry to quantify the difference between two sets of coordinates, such as the positions of atoms in two molecular structures. It calculates the average of the squared differences between corresponding coordinates and then takes the square root of that average, providing a single value that reflects the overall structural dissimilarity between the two data sets. RMSD is particularly useful for assessing the accuracy of molecular modelling and predicting how well a theoretical structure matches an experimental one. The human 70 kda heat shock protein 5 (BIP/GRP78) ATPase (PDBID: 3IUC) in complex with 3-1-BenCarMethInYlPro-Phosphonic acid has shown initial fluctuations at 0.10 ns in the case of protein 1.26 Å and ligand 1.20 Å. While the simulative period complex exhibited stability, and at 100 ns, both protein and ligand reached a deviation of 2.33 Å and 1.79 Å, the complex illustrates stability after ignoring 1ns (Figure 5Aa). The human myosin 9b RHOGAP domain (PDBID: 5C5S) in complex with 3-1-BenCarMethInYlPro-Phosphonic acid, there was an initial slight fluctuation noticed of protein to 1.37 Å, while ligand to 0.95 Å. During simulation, at 100 ns, protein and ligand deviation increased to 5.47 Å and 5.92 Å (Figure 5Ba). The allosteric EYA2 phosphatase inhibitor (PDBID: 5ZMA) in complex with 3-1-BenCarMethInYlPro-Phosphonic acid showed initial fluctuations in the simulation by a gradual stabilisation. The protein and ligand showed 1.78 Å and 2.61 Å, respectively, at 0.10 ns. AT 100 ns, the protein deviation was 3.52 Å, while the ligand deviation was 2.32 Å (Figure 5Ca). The RSK N-terminal kinase domain (PDBID: 6G77) in complex with 3-1-BenCarMethInYlPro-Phosphonic acid deviated in the case of protein to 1.26 Å while in case of ligand to 1.13 Å, respectively. After 100 ns during simulation, both protein and ligand reached deviation of 2.44 Å and 5.30 Å. However, the complex displayed stability after ignoring 1ns (Figure 5Da). The human collapsin response mediator protein-1 (PDBID: 4B3Z) in complex with 3-1-BenCarMethInYlPro-Phosphonic acid, at 0.10 ns, at the beginning was fluctuation observed of protein 0.98 Å and ligand 1.13 Å. However, at 100 ns of simulation, the protein and ligand deviation increased to 1.89 Å and 2.74 Å (Figure 5Ea). Although there were initial fluctuations, the complexes generally attained stability and functioned effectively during the simulation. The variations in protein and ligand structures offer important molecular insights, assisting in thoroughly analysing complex behaviour.

#### 2.5.2. Root Mean Square Fluctuations (RMSF)

The RMSF measures the average deviation of atomic positions in a molecular system over a period, highlighting the flexibility of molecules, and it is crucial in molecular dynamics simulations to understand how atoms move and fluctuate within a structure, aiding in studying protein conformational changes and ligand binding. The human 70 kda heat shock protein 5 (BIP/GRP78) ATPase (PDBID: 3IUC) in complex with 3-1-BenCarMethInYlPro-Phosphonic acid has shown a few fluctuating residues beyond 2 Å are GLY28, GLY134, GLY135, and GLU215, and at the same time, many intermolecular interactions were formed to make the complex stable are ASP34, THR37, THR38, TYR39, ILE61, LYS81, LYS96, TYR175, ASP224, GLY227, GLY228, THR229, GLY255, GLU256, ASP259, GLU293, LYS296, ARG297, GLY364, SER365, ARG367, and ASP391 (Figure 5Ab). The human myosin 9b RHOGAP domain (PDBID: 5C5S) in complex with 3-1-BenCarMethInYlPro-Phosphonic acid has shown a few fluctuating residues beyond 2 Å are PHE109-PRO114, SER126-ALA129, PRO217, GLU218, ASN279-LYS287, LYS291, ILE292, and VAL309-GLU318 and at the same time, many intermolecular interactions were formed to make the complex stable are ALA224, TYR227, LEU230, GLU231, LEU233, GLU235, HIS238, ASN239, LEU241, GLU242, ILE245, PHE246, LEU299, GLU302, GLN303, ARG305, LYS306, TYR307, and LYS310 (Figure 5Bb). The allosteric EYA2 phosphatase inhibitor (PDBID: 5ZMA) in complex with 3-1-BenCarMethInYlPro-Phosphonic acid has shown a few fluctuating residues beyond 2 Å are ASP264, ASN265, ASP297-THR300, ARG303, VAL336-GLN343, SER357-VAL374, VAL398-GLY400, PRO405, and GLU534-GLU536, and at the same time, many intermolecular interactions were formed to make the complex stable are ASP276, GLU277, THR278, ILE279, ILE281, PHE282, HIS283, SER284, THR289, PHE290, ARG293, TYR294, ARG414, LEU417, GLU418, LEU420, THR421, ASP422, LEU423, TRP424, LEU425, THR426, TYR461, ASP502, ILE520, and SER521 (Figure 5Cb). The RSK4 N-terminal kinase domain (PDBID: 6G77) in complex with 3-1-BenCarMethInYlPro-Phosphonic acid has shown a few fluctuating residues beyond 2 Å are VAL51, VAL52, ASP116, VAL118-MET122, GLU227-LYS229, and ALA230-GLY235 and at the same time, many intermolecular interactions were formed to make the complex stable are LYS77, LEU79, GLY80, GLN81, GLY82, SER83, PHE84, GLY85, VAL87, ALA103, LYS105, VAL136, LEU152, PHE154-ASP159, ASP198, LYS200-LEU205, THR215, ASP216, and LYS221 (Figure 5Db). The human collapsin response mediator protein-1 (PDBID: 4B3Z) in complex with 3-1-BenCarMethInYlPro- Phosphonic Acid has shown a few fluctuating residues beyond 2 Å are SER80, GLN81, GLY82, MET83, THR84, ALA85, ALA86, LYS487, PHE489, and GLY490, and at the same time, many intermolecular interactions were formed to make the complex stable are TYR75, LYS78, PRO79, SER80, GLN81, MET83, VAL109, PRO112, ASP140, GLN165, TYR167, TYR170, VAL173, TYR174, HIS198, ARG227, LYS254, THR303, SER304, TYR336, THR349, LEU350, ILE351, GLU353, and GLY354 (Figure 5Eb). The RMSF analysis of protein-ligand complexes involving 3-1-BenCarMethInYlPro-Phosphonic acid revealed specific fluctuating residues. The first complex’s residues GLY28, GLY134, GLY135, and GLU215 exhibited fluctuations beyond 2Å, and conversely, several stable intermolecular interactions were identified, involving residues such as ASP34, THR37, TYR39, LYS81, and others, contributing to complex stability. Similar fluctuation patterns and stabilising interactions were observed in complexes with Human Myosin 9B RhoGAP (PDBID: 5C5S), Allosteric EYA2 Phosphatase Inhibitor (PDBID: 5ZMA), RSK4 N-terminal Kinase Domain (PDBID: 6G77), and Human Collapsin Response Mediator Protein-1 (PDBID: 4B3Z). These findings provide insights into these complexes’ dynamic behaviour and stabilising forces.

#### 2.5.3. Simulation Interaction Diagrams (SID)

**Figure 6:**
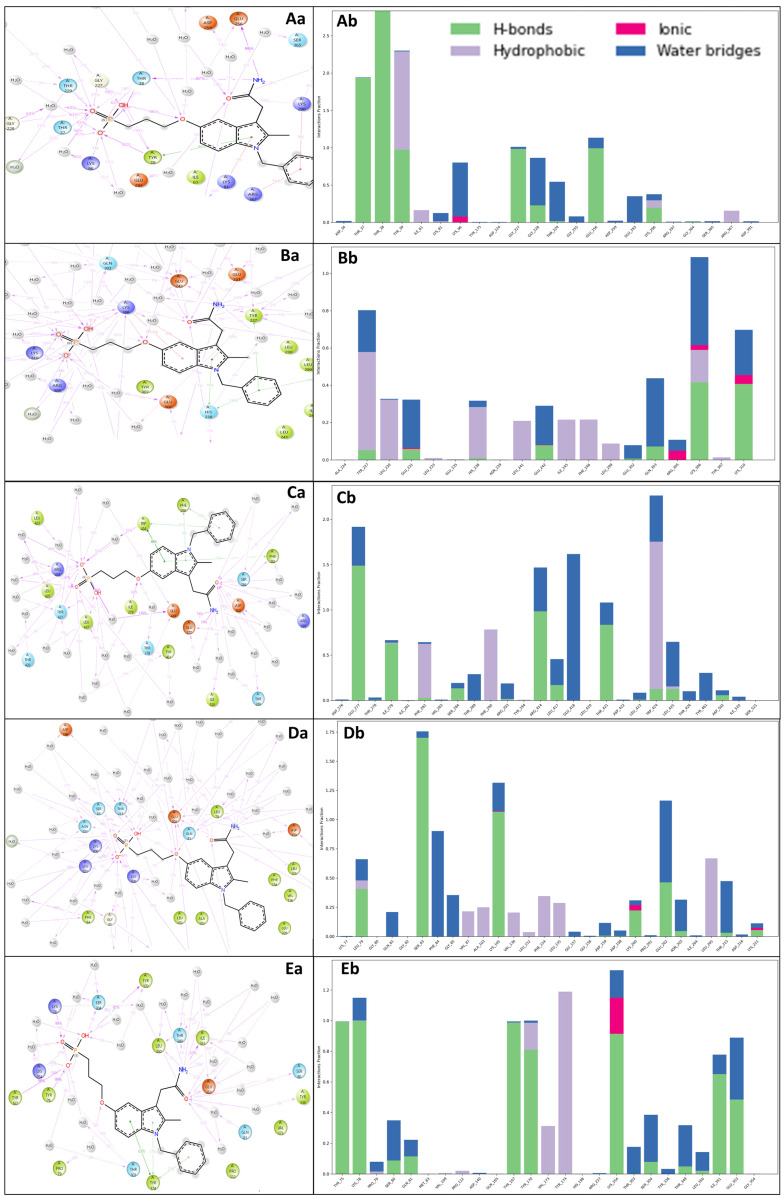
Showing the Simulation Interaction Diagram (SID) of 3-1-BenCarMethInYlPro-Phosphonic Acid in complex with (**Aa**) 3IUC, (**Ba**) 5C5S, (**Ca**) 5ZMA, (**Da**) 6G77 and, (**Ea**) 4B3Z, and the histogram representation of count of the interaction of (**Ab**) 3IUC, (**Bb**) 5C5S, (**Cb**) 5ZMA, (**Db**) 6G77, and (**Eb**) 4B3Z where red colour shows ionic interactions, blue colour shows water bridges, green colour shows H-bonds, and the grey colour shows hydrophobic interactions to make it clear to understand.

The SID, often used in MD simulations, is a graphical representation that illustrates how molecules or atoms interact with each other over time during a simulation. These diagrams help visualise the dynamic behaviour of molecular systems and provide insights into their structural changes and interactions. The interaction of human 70 kda heat shock protein 5 (BIP/GRP78) ATPase (PDBID: 3IUC) in complex with 3-1-BenCarMethInYlPro-Phosphonic acid, there are more than 15 water bridges with water molecules which show stability between molecules in complex. Many hydrogen bonds interact with GLU256 and THR38 along the NH_2_ atom, THR37, THR38, TYR39, and GLY228 along O atoms, and GLY227, THY39, and THR38 along the OH atom. Additionally, a pi–pi stacking contact THR39 and a pi–cation contact ARG367 with the benzene ring of the ligand (Figure 6Aa). The human myosin 9b RHOGAP domain (PDBID: 5C5S) in complex with 3-1-BenCarMethInYlPro-Phosphonic acid produced hydrogen bonding among GLU242 and GLU231 with NH_2_ atom, LYS310, LYS306, GLN303, GLU231 with water molecules, and GLU242 with water molecules along with 4 O atoms. Also, 3 pi–pi stacking along TYR227 and HIS238 with a benzene ring, and 2 pi–cation contact along LYS306 with two benzene rings of the ligand (Figure 6Ba). The allosteric EYA2 phosphatase inhibitor (PDBID: 5ZMA) in complex with 3-1-BenCarMethInYlPro-Phosphonic acid showing interaction with hydrogen bonding among ILE279, ARG414, and TRP424, and THR289, SER284, ARG293, THR421, THR426, LEU425, and LEU423 with water molecules contact along 4 O atoms and LEU417 and THR421 with water molecules along OH atom. Additionally, 5 pi–pi stacking contact TRP424, PHE282, and PHE290 with 3benzene rings of the 3-1-BenCarMethInYlPro-Phosphonic Acid ligand (Figure 6Ca). Interaction between RSK4 N-terminal kinase domain (PDBID: 6G77) in complex with 3-1-BenCarMethInYlPro-Phosphonic acid involves more than 15 water molecules forming water bridges. There are many hydrogen bonds among VAL87 and LEU79 with water molecules, LYS105 and THR215 with water molecules, LYS105, SER83, and PHE84 with water molecules contact along 4O atoms, GLU202 and ASN203 with water molecules along OH atom, and LEU79 and GLN81, ASP159 with water molecules along NH_2_ atom (Figure 6Da). The human collapsin response mediator protein-1 (PDBID: 4B3Z) in complex with 3-1-BenCarMethInYlPro-Phosphonic acid involves 16 water bridges with water molecules. Interaction with many hydrogen bonds among GLU353, SER80, TYR75, TYR167, and PRO79 with water molecules with 4O atoms, SER304, TYR170, and LYS254 along OH atom. Also, 3 pi–pi stacking contacts TYR174 with the benzene ring of the ligand (Figure 6Ea). The SID results reveal intricate molecular interactions in the complexes formed between 3-1-BenCarMethInYlPro-Phosphonic acid and the target proteins. In the case of human 70 kda heat shock protein 5 (BIP/GRP78) ATPase (PDBID: 3IUC), numerous water bridges are observed, indicating stable connections between molecules within the complex. Hydrogen bonds play a significant role, with interactions involving amino acids such as GLU256, THR38, THR37, TYR39, and GLY228, enhancing the complex’s stability. Furthermore, pi–pi stacking and pi–cation contacts contribute to ligand binding. Similarly, the complex involving human myosin 9b RHOGAP domain (PDBID: 5C5S) exhibits extensive hydrogen bonding, water-mediated interactions, and pi–pi stacking contacts, underscoring the robustness of the binding. The allosteric EYA2 phosphatase inhibitor (PDBID: 5ZMA) demonstrates multiple hydrogen bonds, water bridges, and pi–pi stacking interactions, emphasising the structural integrity of the complex. The RSK4 N-terminal kinase domain (PDBID: 6G77) also exhibits substantial water bridges and hydrogen bonds, highlighting the intricate network of interactions supporting the complex. The human collapsin response mediator protein-1 (PDBID: 4B3Z) complex is characterised by numerous water bridges, hydrogen bonds, and pi–pi stacking contacts, further confirming the stability and reliability of the binding interface. These detailed interaction profiles provide valuable insights into the MD of these complexes, provided the potential as drug candidates, and the count of interactions is shown in the histogram in Figure 6Ab,Bb,Cb,Db,Eb.

## 3. Discussion

In our study, we comprehensively analysed protein–ligand interactions and structural properties to evaluate the potential of 3-1-BenCarMethInYlPro-Phosphonic acid as a multitargeted inhibitor for lung cancer-related proteins. Our investigation began with the meticulous preparation and validation of protein structures for molecular docking. We retrieved 3D structures of target proteins, including PDBID: 3IUC, 5C5S, 5ZMA, 6G77, and 4B3Z, from the RCSB Protein Data Bank [25,26,27,28,29]. These structures underwent rigorous pre-processing within Maestro’s Protein Preparation Workflow (PPW), encompassing side chain completion, loop modelling, bond order assignment, hydrogen removal, and disulfide bond formation. This meticulous preparation ensured structural integrity, a prerequisite for reliable molecular docking studies. We employed Ramachandran plot analysis to validate the protein structures further, which assessed the dihedral angles of amino acid residues. The distribution of residues within the favourable regions of the plot indicated the correctness and suitability of these proteins for subsequent molecular docking studies. This validation process provided a crucial foundation for our investigation, ensuring the reliability of our results. Subsequently, we delved into the protein–ligand interaction analysis using induced fit docking, focusing on 3-1-BenCarMethInYlPro-Phosphonic acid as a potential drug candidate. Our molecular interaction analysis diagram depicted the interactions between the adaptive protein structures and the ligands. The detailed results showcased promising docking and MMGBSA scores for each complex, indicating strong binding affinities. Hydrogen bonds played a significant role in these interactions, forming crucial connections between the ligand and key amino acid residues. Additionally, pi–pi stacking and pi–cation contacts further stabilised the complexes, highlighting the potential of 3-1-BenCarMethInYlPro-Phosphonic acid as a multitargeted inhibitor of lung cancer-related proteins.

The computational analysis of 3-1BCMIYPPA has yielded valuable insights into its electronic and vibrational properties. With 557 canonical orbitals, the molecule exhibits a HOMO energy level of −0.053769 Hartree and a LUMO energy level of −0.006937 Hartree, signifying its electronic structure. A dipole moment of 37.3867 Debye indicates its polarity. Vibrational analysis reveals a stable molecule with frequencies ranging from 25.961 cm^−1^ to 3739.59 cm^−1^ and no negative frequencies. The zero-point energy is 268.71 kcal/mol. Thermodynamic properties at standard conditions include entropy (171.653 kcal/mol/K), enthalpy (17.239162 kcal/mol), free energy (−33.93919 kcal/mol), internal energy (16.646677 kcal/mol), and heat capacity (105.347 Kcal/mol). ESP analysis shows charge distribution, while ALIE analysis elucidates noncovalent interactions. We assessed the drug-like characteristics of 3-1-BenCarMethInYlPro-Phosphonic acid for the pharmacokinetics using the QikProp tool. The compound displayed favourable properties, including small molecular size, compliance with Lipinski’s Rule of Three, and high human oral absorption potential. These characteristics and the acceptable number of hydrogen bond donors and acceptors suggest its potential suitability for drug development. Physicochemical properties, such as low dipole moment, polarisability, and solvent-accessible surface area, align with drug development criteria. However, a negative CNS score indicates potential inactivity in the central nervous system, warranting further investigation. The molecular interaction fingerprints reinforced the stability of the complexes. Specific amino acid residues, including ILE, GLY, VAL, TYR, LEU, and GLN, played pivotal roles in anchoring the ligand within the binding site. Moreover, THR, SER, PHE, ARG, PRO, LYS, HIE, ASP, ASN, ALA, TRP, MET, HIS, and ASP residues contributed to the overall stability of these complexes. These robust molecular interactions collectively created a reliable binding interface, further highlighting the potential of 3-1-BenCarMethInYlPro-Phosphonic acid as an effective inhibitor.

In our study, we conducted extensive Molecular Dynamics (MD) simulations to gain insights into the behaviour and stability of protein-ligand complexes involving 3-1-BenCarMethInYlPro-Phosphonic acid as a potential multitargeted inhibitor for lung cancer-related proteins. We performed simulations for five different complexes, and the results shed light on the dynamic behaviour of these complexes. The Root Mean Square Deviation (RMSD) analysis is a critical measure in structural biology and computational chemistry that quantifies the difference between two sets of atomic coordinates, assessing structural dissimilarity. In our simulations, we observed initial deviations in the complexes, especially in the case of the human 70 kda heat shock protein 5 (BIP/GRP78) ATPase complex. However, these complexes eventually reached stability, a positive sign for their reliability in drug development efforts. The Root Mean Square Fluctuations (RMSF) analysis allowed us to understand the flexibility of the complexes’ constituent atoms. Several residues in these complexes exhibited fluctuations beyond 2 Å, indicating their dynamic nature. This analysis pinpointed specific flexible residues within the complexes, such as GLY28, GLY134, GLY135, and GLU215 in the BIP/GRP78 ATPase complex. However, these fluctuations were balanced by stabilising interactions like hydrogen bonds with ASP34, THR37, THR38, TYR39, and LYS81. Similarly, in the Myosin 9b RHOGAP complex, residues like PHE109-PRO114 exhibited fluctuations beyond 2 Å, but interactions involving ALA224, TYR227, LEU230, GLU231, and others-maintained stability. In the Allosteric EYA2 Phosphatase Inhibitor complex, residues ASP264, ASN265, ASP297-THR300 fluctuated, but hydrogen bonds with ILE279, ARG414, TRP424, and others, plus pi–pi stacking contacts with TRP424, PHE282, and PHE290, ensured structural integrity. In the RSK4 N-terminal Kinase Domain complex, fluctuations in residues like VAL51 were offset by hydrogen bonds with LYS77, LEU79, GLY80, and others. Finally, the Collapsin Response Mediator Protein-1 complex exhibited fluctuations in residues such as SER80, GLN81, GLY82, MET83, THR84, ALA85, ALA86, LYS487, PHE489, and GLY490, but stabilising interactions with TYR75, LYS78, PRO79, and pi–pi stacking contacts with TYR174 maintained stability. The SID visualisations provide a detailed picture of how molecules or atoms interacted within the complexes throughout the simulations. In all five complexes, we observed various interactions, including hydrogen bonds, water bridges, pi–pi stacking, and pi–cation contacts. These interactions are essential for maintaining the stability of the complexes and play a significant role in ligand binding. For example, in the human 70 kda heat shock protein 5 (BIP/GRP78) ATPase complex, we identified more than 15 water bridges and numerous hydrogen bonds. These interactions highlight the stable connections between molecules within the complex. Additionally, pi–pi stacking and pi–cation contacts further contribute to the ligand’s binding affinity. Similarly, in the complex with the human myosin 9b RHOGAP domain, we observed extensive hydrogen bonding, water-mediated interactions, and pi–pi stacking contacts. These findings emphasise the robustness of the binding and the potential of 3-1-BenCarMethInYlPro-Phosphonic acid as an inhibitor. The allosteric EYA2 phosphatase inhibitor complex displayed multiple hydrogen bonds, water bridges, and pi–pi stacking interactions. These structural features underscore the stability and integrity of the complex. The RSK4 N-terminal kinase domain complex also exhibited substantial water bridges and hydrogen bonds, illustrating the intricate interaction network that supports the complex’s stability. In the complex involving the human collapsin response mediator protein-1, we observed numerous water bridges, hydrogen bonds, and pi–pi stacking contacts, further confirming the stability and reliability of the binding interface. Our MD simulations provided valuable insights into these protein–ligand complexes’ dynamic behaviour and stabilising forces.

Comparatively, previous endeavours in multitargeted drug design for lung cancer have often faced challenges in achieving a delicate balance between efficacy and safety. The intricate network of signalling pathways and molecular interactions in lung cancer demands a nuanced approach. In this context, the comprehensive analysis presented in our study validates the potential of 3-1-BenCarMethInYlPro-Phosphonic acid and highlights the significance of a systematic and detailed investigation. The success story of 3-1-BenCarMethInYlPro-Phosphonic acid as a multitargeted inhibitor is underscored by its robust binding affinities, stable interactions, and favourable pharmacokinetic profile. These attributes position it as a promising candidate for further experimental validation. Compared to traditional single-target drugs, the multitargeted approach offers the advantage of addressing the complexity of lung cancer at various levels, potentially enhancing therapeutic outcomes. The findings from our molecular dynamics simulations further contribute to the success narrative by providing valuable insights into the dynamic behaviour and stabilising forces of protein-ligand complexes. The observed stability during simulations is a positive indicator of the compound’s potential as a drug candidate. This success story reflects the evolving paradigm in drug design, emphasising the importance of understanding the dynamic interplay between proteins and ligands. Our study not only positions 3-1-BenCarMethInYlPro-Phosphonic acid as a promising multitargeted inhibitor for lung cancer-related proteins but also contributes to the broader discourse on the success and challenges of multitargeted drug design in the context of lung cancer. The intricate details uncovered through our analyses add depth to the understanding of molecular interactions, structural properties, and dynamic behaviour, paving the way for future advancements in drug discovery for lung cancer. These findings support the potential of 3-1-BenCarMethInYlPro-Phosphonic acid as a promising multitargeted inhibitor for lung cancer-related proteins. The stability exhibited by these complexes during the simulations is encouraging for their potential as drug candidates, and further experimental validation is warranted based on these promising results. Our findings provide valuable insights into the MD simulation of these complexes and lay the groundwork for potential drug development efforts against lung cancer. The analysis of protein–ligand interactions, structural properties, pharmacokinetics, and molecular dynamics simulations underscores the potential of 3-1-BenCarMethInYlPro-Phosphonic acid as a promising multitargeted inhibitor for lung cancer-related proteins. This compound’s strong binding affinities, stable interactions, and drug-like characteristics make it a valuable candidate for further experimental validation.

## 4. Methods and Materials

**Figure 7:**
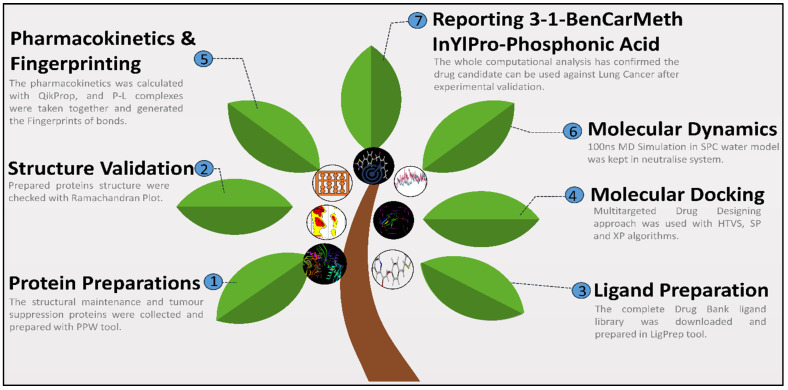
Graphical Abstract or the study flow to identify Lung cancer’s multitargeted inhibitor.

The complete methods can be clearly understood by the graphical abstract or the flow of the study (Figure 7), and the detailed methods are as follows:

### 4.1. Ligand and Protein Preparation and Structural Validation

The Drug Bank database (ligand library) was downloaded from (https://go.drugbank.com/) (accessed on 10 January 2023) after getting the permission and approved account for academic use, and prepared using the Schrodinger Maestro’s LigPrep tool, where we kept the criteria to filter the compounds beyond 500 atoms and used the OPLS4 forcefield [33,34,35,36]. The ionisation was observed to generate the possible states at the target pH of 7 ± 2 and used the Epik and added metal binding conditions and included the selected original states [37]. The Desalt and generated tautomers were kept, stereoisomers were used to retain specified chiralities and generate 32 ligands per compound, and the output files were saved in structure data format. For the 3D structures of the protein, we went through various literature to identify the crucial proteins and then searched in the RCSB database (http://rcsb.org/) [38,39]. We found human 70 kda heat shock protein 5 (BIP/GRP78) ATPase (PDBID: 3IUC) [25], human myosin 9b RHOGAP domain (PDBID: 5C5S) [26], allosteric EYA2 phosphatase inhibitor (PDBID: 5ZMA) [27], RSK4 N-terminal kinase domain (PDBID: 6G77) [28], and human collapsin response mediator protein-1 (PDBID: 4B3Z) [29] and prepared them in Protein Preparation Workflow (PPW) tool in Maestro [34,40,41]. We found that 3IUC contains ligands, Chain A, Chain B of protein, solvents, and metals/ions, 5C5S contains Chain A, B, C, and D of the protein and solvents, 5ZMA contains Chain A, B, and C of protein and ligand, 6G77 contains Chain A and B of the protein, ligands, solvents, and metals/ions, and 4B3Z contains only protein chains of A, B, C, and D. In PPW, we capped the termini, filled in the missing side chains, filled in the missing loops using Prime, assigned bond orders to CCD database, deleted hydrogens, created disulphide bonds, zero bond orders to metals, converted selenomethionines to methionine, and generated possible hetero states using Epik at pH 7.4 ± 2 [37]. Sample water orientation and crystal symmetry were used as all the proteins had 3D crystal structures, minimised hydrogen of altered species, and optimised proteins using PROPKA [42]. Converge heavy atoms to RMSD 0.30 Å and delete the water beyond 5 Å to ligand and minimise the protein OPLS4 force field [36]. All the proteins were prepared using the same parameters and kept only clean Chain A of the proteins in each condition. Further, we manually checked each structure and analysed the Ramachandran plot to confirm the protein’s correctness and fitness for the docking studies.

### 4.2. Receptor Grid Generation (RGG) and Molecular Docking

In docking with Glide, the grid is crucial in predicting the binding affinity between a ligand (small molecule) and a target protein. It represents the 3D space around the protein where the ligand can bind [43]. Glide uses a grid-based scoring function to evaluate different ligand conformations within this grid, helping identify the most favourable binding pose. The grid aids in accurately predicting ligand–protein interactions and guiding drug discovery efforts. Maestro’s Receptor Grid Generation (RGG) tool was used to generate the grids on each protein, and we have kept the scaling factor of 1.0 and partial charge cutoff of 0.25 [34,43,44]. In the site tab, the docked ligand is confined to the enclosing box and displayed in the box to fix appropriately on the proteins. The centroid of the specified residues was selected as an option and defined all atoms to generate a proper grid on the complete protein structure and the box size was adjusted to fit the grid properly on the protein, and the jobs were run to produce the grid file in zipped format for the next step of the docking studies. The molecular docking-based screening was performed using the Virtual Screening Workflow (VSW) tool, and in the input tab, we browsed the prepared library of the ligand. In the filtering tab, we used the QikProp to generate the compounds’ pharmacokinetic properties and the properties were passed to Lipinski’s rules to filter further [45,46,47,48]. We skipped the preparation step as our ligand was already prepared, and in the receptor tab, we browsed each receptor grid. We used the Epik state penalties for docking in the docking tab and wrote interaction scores for residues within 12 Å of the grid centre [37]. The ligand scaling of van der Waals radii for nonpolar atoms was kept with a scaling factor of 0.80, and the partial charge cutoff was kept at 0.15. Furthermore, the screening was kept with the High Throughput Virtual Screening (HTVS), Standard Precision, and Extra Precise (XP) followed by pose filtering with Molecular Mechanics based Generalised Born Surface Area (MM\GBSA) studies [34,43,44]. The whole filtered compound’s library was passed to HTVS for screening, and only the top 10% of HTVS data was passed to SP, and the top 10% of SP data was passed to the XP screening algorithm. In the HTVS, the flexible docking was kept, and penalise the non-planar conformations performed post-docking minimisation, generated up to 1 pose per compound state, and then retaining all states were kept and in the SP docking methods, the same parameters were kept except the retaining all good scores was checked this time. Further, in the XP, we kept generating up to four poses per compound, retaining only best scoring states, and 100% of the data of XP was passed to MM\GBSA for computing binding free energy and filtering the top poses for the analysis by following these complex screening methods we were able to reduce the computational cost and enhanced sampling of the compounds.

### 4.3. Pharmacokinetics and QM-Based DFT Studies

Pharmacokinetic properties refer to how the body absorbs, distributes, metabolises, and eliminates a drug. These properties include absorption rates, distribution within tissues, enzyme metabolism, and excretion through various routes, such as the kidneys. Understanding a drug’s pharmacokinetics is crucial in determining its dosing regimen, efficacy, and potential side effects. It helps ensure that the drug reaches the target site in the body at the right concentration and for the required duration to achieve therapeutic effects. The QikProp tool was used to compute the pharmacokinetic properties of the compound 3-1-BenCarMethInYlPro-Phosphonic Acid and filtered with Lipinski’s rules and compared with the standard values to make it clear to understand if the reporting compound is suitable as a drug candidate or not [34,45]. The optimisation process refines the geometry of molecules, predicting their most stable and energetically favourable configurations, and is valuable for understanding molecular properties interactions and assisting in the design of novel drugs by fine-tuning molecular structures for improved binding to target proteins. We employed the Jaguar Optimization tool to optimise our chosen ligand molecule, selecting the default B3LYP-D3 theory with a 6-13G*** basis set for Density Functional Theory [34,49]. The SCF spin treatment was automatic, and the initial guess used quick and atomic overlap in the SCF Table Convergence criteria included a maximum of 48 iterations, an energy change of 5 × 10^−5^ Hartree, and an RMS density matrix change of 5 × 10^−6^. We maintained stability with an SCF level shift of 0.0 Hartree and no thermal smearing, employing the DIID convergence scheme. We allowed 100 steps in the optimisation tab, using Schlegel’s guess and redundant internal coordinates for the initial hessian. Property calculations encompassed all properties, including vibration frequencies at thermochemistry conditions of 1.0 atm pressure and an initial temperature of 298.15 K in Kal/mol units. Surface calculations considered electrostatic potential with no specified box size and an average local ionisation energy of 5 pts/Å Kcal/mol. Noncovalent interactions were explored with a grid density of 20 pts/Å, focusing on density and spin density. Molecular Orbitals were defined from α HOMO-0 to LUMO+0 with two orbitals and β HOMO-0 to LUMO+0 with the same two orbitals. Solvation was addressed using the PBF model in a water solvent medium, and the output was saved in Gaussian format. For result analysis, we utilised the QM convergence monitor tool in Jaguar [34].

### 4.4. Interaction Fingerprints

Molecular Interaction Fingerprints represent molecular interactions between a molecule and its environment and capture how a molecule interacts with other molecules, such as proteins or ligands, by encoding specific structural and chemical features. These fingerprints are used in drug discovery and computational chemistry to identify and characterise potential binding interactions, design new drugs, and understand molecular behaviours in complex systems [2,6,15,24,48]. The molecular interaction fingerprints were generated in Maestro using the Interaction Fingerprinting tool, where we selected the protein-ligand complex in type, and any contact type was chosen and aligned all the sequences while keeping PDBID: 4B3Z as the reference sequence and generated the fingerprints and displayed the interaction matrix by keeping all default properties [34]. In the interaction matrix panel, any contact was selected, and coloured the main plot with residues from N to C terminal by the sequence number and kept only interacting residues and showed the count of ligand interaction and count of interacting residues to make it clear to understand which type of residues are interacting more.

### 4.5. Molecular Dynamics (MD) Simulation

The MD Simulation is a crucial tool to analyse the stability of the protein–ligand complexes, and it was performed using the Desmond package available freely for academic use from https://www.deshawresearch.com/, developed by the D. E. Shaw Research [2,32,50,51]. The best-docked pose of proteins in complex with 3-1-BenCarMethInYlPro-Phosphonic acid was selected for the MD simulation, which was performed in two steps: one is in preparation of the system and the second is production run and analysis. The system builder tool selected a predefined SPC water model [52] with orthorhombic boundary conditions in the buffer with a distance of 10 × 10 × 10 Å and the boundary box fitting correctly on the complex. The salt and ion placements were excluded within 20Å and added no ions in 3IUC, 7 Na^+^ in 5C5S and 5ZMA or 4 Cl^−^ in 6G77 and 8 Na^+^ in 4B3Z to neutralise them and used the OPLS4 force field to run the jobs [36]. The Molecular Dynamics panel was used for the production run. The prepared system file was loaded to the panel, and in the simulation, 100 ns was defined, with a recording interval of 100 ps kept at an energy level of 1.2 that generated 1000 frames per simulation. At 300 K temperature, the NPT ensemble class and 1.01325 bar pressure were used, and the system was relaxed before the simulation [53]. Further, we have used the simulation interaction diagram (SID) tool to analyse the trajectories of the MD simulation and exported the figure for better representation.

## 5. Conclusions

Our extensive investigation into the potential of 3-1-BenCarMethInYlPro-Phosphonic acid as a multitargeted inhibitor for lung cancer proteins has several crucial findings. We rigorously prepared and validated protein structures, ensuring their structural integrity for reliable molecular docking studies and Ramachandran plot analysis confirmed the suitability of these structures. The molecular docking analysis revealed strong binding affinities between 3-1-BenCarMethInYlPro-Phosphonic Acid and target proteins, supported by hydrogen bonds, pi–pi stacking, and pi–cation interactions, and it suggests the compound’s potential as an effective inhibitor of lung cancer-related proteins. The pharmacokinetic assessments indicated favourable drug-like characteristics, high oral absorption potential, and compliance with key criteria. Molecular dynamics simulations demonstrated the stability of these complexes over a 100ns timeframe, reinforcing their suitability for further analysis. Our study provides compelling evidence for the potential of 3-1-BenCarMethInYlPro-Phosphonic acid as a multitargeted inhibitor for lung cancer-related proteins., offering hope for improved therapies in the fight against lung cancer.

## Figures and Tables

**Table 1:** Showing the Docking Score with MM\GBSA scores (Kcal/mol) and other important computations produced after multitargeted molecular docking studies.

SNo	PDBID	Docking Score	MMGBSA dG Bind	Ligand Efficiency Sa	Ligand Efficiency Ln	Evdw	Ecoul
1	3IUC	−9.02	−39.68	−2.05	−9.09	−42.64	−26.63
2	5C5S	−5.83	−36.30	−1.88	−8.31	−31.05	−14.89
3	5ZMA	−7.47	−7.56	−0.39	−1.73	−30.30	−6.92
4	6G77	−10.66	−50.14	−2.59	−11.48	−38.69	−22.65
5	4B3Z	−9.71	−43.56	−2.25	−9.97	−21.72	−19.85

**Table 2:** Showing the Pharmacokinetics prediction of 3-1-BenCarMethInYlPro-Phosphonic acid against standard values of QikProp.

Descriptor	Standard Values	3-1-BenCarMethInYlPro-Phosphonic Acid	Descriptor	Standard Values	3-1-BenCarMethInYlPro-Phosphonic Acid
Type	N/A	Small	glob	0.75–0.95	0.7929173
#amidine	0	0	EA(eV)	−0.9–1.7	−0.03
HumanOralAbsorption	-	1	#metab	1–8	5
#ringatoms	-	15	dipole	1.0–12.5	3.074
#in34	-	0	QPlogKhsa	−1.5–1.5	−0.703
#in56	-	15	QPpolrz	13.0–70.0	40.839
#noncon	-	0	mol MW	130.0–725.0	416.413
#nonHatm	-	29	#NandO	2–15	7
Jm	-	0.014479409	CNS	−2 (inactive), +2 (active)	−2
QPPCaco	<25 poor, >500 great	1.58	accptHB	2.0–20.0	8.25
QPPMDCK	<25 poor, >500 great	1.55	QPlogPo/w	−2.0–6.5	2.523
%HumanOralAbsorption	>80% = high, <25% = poor	45.275	QPlogBB	−3.0–1.2	−2.697
#amine	0–1	0	SASA	300.0–1000.0	722.945
#acid	0–1	2	QPlogPC16	4.0–18.0	14.715
#amide	0–1	1	QPlogPw	4.0–45.0	18.956
#rotor	0–15	11	volume	500.0–2000.0	1290.483
#rtvFG	0–2	0	QPlogS	−6.5–0.5	−3.356
#stars	0–5	0	CIQPlogS	−6.5–0.5	−4.496
AcxDN^0.5^/SA	0.0–0.05	0.0228233	PSA	7.0–200.0	128.095
dip^2^/V	0.0–0.13	0.0073203	FISA	7.0–330.0	245.723
SAfluorine	0.0–100.0	0	IP(eV)	7.9–10.5	8.031
WPSA	0.0–175.0	3.379	QPlogKp	−8.0–−1.0	−4.103
SAamideO	0.0–35.0	34.445	QPlogPoct	8.0–35.0	24.13
PISA	0.0–450.0	252.499	QPlogHERG	concern below −5	−1.098
donorHB	0.0–6.0	4	RuleOfThree	maximum is 3	1
FOSA	0.0–750.0	221.344	RuleOfFive	maximum is 4	0

## Data Availability

The manuscript provides all the data in figures and tables.
